# Salient objects dominate the central fixation bias when orienting toward images

**DOI:** 10.1167/jov.21.8.23

**Published:** 2021-08-25

**Authors:** Christian Wolf, Markus Lappe

**Affiliations:** 1 Institute for Psychology, University of Muenster, Münster, Germany; 2 Institute for Psychology, University of Muenster, Münster, Germany

**Keywords:** global effect, center-of-gravity, center bias, natural scenes, visual salience, figure-ground

## Abstract

Short-latency saccades are often biased toward salient objects or toward the center of images, for example, when inspecting photographs of natural scenes. Here, we measured the contribution of salient objects and central fixation bias to visual selection over time. Participants made saccades to images containing one salient object on a structured background and were instructed to either look at (i) the image center, (ii) the salient object, or (iii) at a cued position halfway in between the two. Results revealed, first, an early involuntary bias toward the image center irrespective of strategic behavior or the location of objects in the image. Second, the salient object bias was stronger than the center bias and prevailed over the latter when they directly competed for visual selection. In a second experiment, we tested whether the center bias depends on how well the image can be segregated from the monitor background. We asked participants to explore images that either did or did not contain a salient object while we manipulated the contrast between image background and monitor background to make the image borders more or less visible. The initial orienting toward the image was not affected by the image-monitor contrast, but only by the presence of objects—with a strong bias toward the center of images containing no object. Yet, a low image-monitor contrast reduced this center bias during the subsequent image exploration.

## Introduction

Human vision is characterized by the foveated nature of our visual system. Whereas the central part of the visual field, the fovea, allows humans to process objects with high scrutiny, acuity in the periphery declines rapidly with increasing eccentricity (for reviews, see Strasburger et al., 2011; Stewart et al., 2020). As a consequence, what we visually process and which details escape our awareness often critically depend on which objects or regions of the visual scene our oculomotor system selects for high-acuity visual processing. This sequential selection-and-sampling process is achieved by saccadic eye movements. Where we saccade to can be determined by a target's saliency, that is, low-level aspects such as a target's luminance contrast (for review, see Itti & Koch, 2001), high-level aspects such as our behavioral goals (for reviews, see Schütz et al., 2011; Tatler et al., 2011; Wolf & Lappe, 2021), and by an individual's history of preceding oculomotor selections (for reviews, see Awh et al., 2012; Le Pelley et al., 2016).

Stimuli that suddenly appear in our visual field are particularly successful in capturing gaze. They can cause an overt orienting response by means of a saccade or, if the saccade is inhibited, a covert shift of attention (Posner, 1980; Sokolov, 1990) that is accompanied by further physiological changes, for example, changes in pupil size (for review, see Wang & Munoz, 2015). Salient stimuli that appear in spatial proximity to a designated saccade target can bias saccade endpoints to a location in between the target and the distracting stimulus (Findlay, 1982). This bias, referred to as center-of-gravity response or as the global effect (for review, see Vitu, 2008; van der Stigchel & Nijboer, 2011), depends on the spatial distance between target and distractor (Walker et al., 1997) as well as on the temporal distance between distractor onset and saccade onset (Ottes et al., 1985; Coëffé & O'Regan, 1987; Heeman et al., 2014). Center-of-gravity responses do not only occur for saccades to two or more items (Fehd & Seiffert, 2008), but also when making saccades to one single target (Vishwanath & Kowler, 2003; Bindemann et al., 2009; Nuthmann & Henderson, 2010). Center-of-gravity responses caused by a distractor are said to arise from averaging across multiple possible saccade vectors and are strongest for saccades that are initiated in a time window approximately 100–300 ms after distractor onset. Given that reaction times of saccades can vary substantially from one trial to the next, even when the response is made to the same stimulus configuration and with the same task at hand (for review, see Sumner, 2011), the bias caused by the distractor in a particular trial can thus be large or small depending on the saccade latency in a trial.

A similar temporal dependency of saccade endpoints has been observed for saccades in response to a single spatially extended target that contained a high-salient region and a low-salient region, when the low-salient region was associated with a reward (Schütz et al., 2012; Wolf & Lappe, 2020). Early saccades were biased toward high salience and only later saccades could be governed by voluntary control and successfully landed in the rewarded region (see also Ludwig & Gilchrist, 2002; van Zoest et al., 2004; van Heusden et al., 2021). This transition from salience to voluntary control was shown to depend on the time it takes to inhibit a response toward the salient region rather than on deliberate planning of saccades into the rewarded region (Wolf & Lappe, 2020). Consistent with the time course of center-of-gravity responses, this saliency bias was strongest for stimulus onsets approximately 100–300 ms before the saccade. However, inhibition of saccades to the salient region could be achieved by previewing the target. A similar observation has recently been made for two distinct targets (Arkesteijn et al., 2020), depending on the eccentricity of stimuli (Walker et al., 1997; Wolf & Lappe, 2020; van Heusden et al., 2021). Thus, whether a saccadic eye movement is directed toward a target that is visually salient because of its low-level properties or toward a target that is attractive because of high-level aspects (e.g., a reward or a behavioral goal), depends on when the saccade is initiated.

This is also true for images of natural scenes (Parkhurst et al., 2002; Anderson et al., 2015; but see Tatler et al., 2005). In the study by Anderson et al. (2015) participants inspected images where one half of the image had an increased or a decreased luminance contrast. Early initial saccades following image onset were predominantly directed toward the side with a higher contrast, whereas delayed initial saccades and all subsequent saccades were more evenly distributed across the two image regions (Anderson et al., 2015). When inspecting images, another bias can be observed that depends on time (Rothkegel et al., 2017; Peacock et al., 2020) and is particularly pronounced for early responses following image onset—the central fixation bias. This central fixation bias or center bias describes the tendency to preferably fixate locations close to the image center and has been reported for a variety of stimuli and for a variety of tasks (Mannan et al., 1996; Tatler, 2007; Tseng et al., 2009; Bindemann, 2010; Zelinsky, 2012). Proposed explanations for the center bias covered low-level and high-level influences (Tatler, 2007; Zelinsky, 2012). For example, one prominent explanation for the central fixation bias was that it arises because of a central pretarget fixation marker (such that gaze is centered on the image when it appears) and the tendency to only make small saccades. Tatler (2007) refuted this explanation by showing that initial saccades directed gaze toward the center even when the fixation marker was displaced (Tatler, 2007; Tseng et al., 2009). He suggested that the image center might constitute a strategically advantageous location when starting to explore an image or that the initial orienting toward the image center might help to rapidly extract the gist of a scene. Rothkegel et al. (2017) showed that the initial orienting toward the image center depends on time and can be reduced by delaying the initial saccade toward the image (Rothkegel et al., 2017; Peacock et al., 2020). This temporal dependency suggests that an explanation for the central fixation bias purely in terms of a strategical advantage is unlikely (Rothkegel et al., 2017), especially since voluntary/strategic control of eye movements is particularly pronounced for long-latency saccades.

It is still unclear how properties and content of images contribute to the central fixation bias. Whereas Tatler (2007) found that the central fixation tendency was comparably strong when free viewing images with salient image features in the center or in the image periphery, Tseng et al. (2009) found that it correlated with a subjective rating of how centered interesting elements were arranged in short movie clips. This inconsistency might either be related to the differences between static images and videos. Or it might be related to the fact that subjective ratings of interesting elements do not reflect low-level properties but are more strongly related to high-level aspects such as the presence of objects, although object locations and low-level image salience correlate (Einhäuser, Spain, & Perona, 2008). High-level aspects were in turn proposed to be the primary targets of attentional selection and to mediate the effects of salience (Nuthmann & Henderson, 2010; Pajak & Nuthmann, 2013; Henderson & Hayes, 2017; Nuthmann et al., 2020). The presence of salient objects can contribute to central fixation tendencies because of the way images are typically taken—with the salient/relevant object located in the center (photographer bias; Tatler et al., 2005; Schumann et al., 2008; Tseng et al., 2009).

The aim of the present work was to reveal whether the initial orienting toward images and particularly the bias toward the image center is purely strategic or whether the orienting toward images is involuntarily biased toward the center of images. Moreover, we aimed to determine whether any observed involuntary bias is due to the presence of salient objects in the center of the image or due to the image center being the center, i.e., because it forms the center of gravity of the image outline. To test this, we conducted Experiment 1 where we measured endpoints of saccades made toward images containing one salient object vertically displaced from the image center. Across different blocks participants were instructed to saccade to different parts of the image: the center, the object, or midway between. In all three conditions looking at the instructed location could be achieved by making purely horizontal saccades. This was made explicit to participants. We systematically changed how the images were vertically located and/or vertically cropped to assure a vertical offset between the salient object and image center. This allowed attributing any systematic vertical deviation in saccade endpoints to either the salient object or the image center, depending on the experimental condition. In a second experiment, we tested how the central fixation bias is affected by the degree to which one can segregate the image borders from the monitor background. Therefore, we asked participants to explore images while the contrast between image background and screen background was low or high and while images either contained a salient object or not.

## Experiment 1

Experiment 1 aimed to reveal whether the initial orienting toward an image is automatically biased toward its center. Therefore, we measured the endpoints of saccades that were made in response to an appearing image. Each image contained one salient object on a structured background. We analyzed endpoints as a function of saccadic reaction time and reconstructed these time courses with high temporal precision. Critically, in the images the salient object and the image center were vertically displaced, and in different blocks we instructed participants to either make saccades toward the (i) image center, (ii) the salient object, or (iii) to a cued location in between the two. In all conditions this could be achieved by making purely horizontal saccades and this information was explicitly provided to participants. This facilitated making saccades to the instructed location and allowed us to attribute any systematic vertical bias in the time course of endpoints to (i) the salient objects or (ii) the image center. The third condition (iii) allowed us to reveal which of the two biases dominated if they exert a pull in different directions. A systematic deviation in vertical saccade endpoints toward the salient object in the *look at image center* condition would be indicative of a center-of-gravity bias caused by the salient object. On the other hand, a systematic deviation in vertical saccade endpoints toward the image center in the *look at object* condition would reveal an involuntary scene-dependent center bias that goes beyond the location of salient objects in the image.

### Methods

#### Participants

We collected data of 18 individuals (four male, 14 female; age range: 18–47 years, median age: 23 years). Participants were undergraduate students from the University of Muenster (*N* = 14) or lab members (*N* = 4). All participants had normal or corrected-to-normal vision and were naïve with regard to the purpose of the experiment. Undergraduate students were reimbursed with course credit or 8 €/hour. All participants provided written informed consent before testing and experiments were conducted in accordance with the Declaration of Helsinki.

#### Setup

We presented stimuli on an Eizo FlexScan 22-inch CRT monitor (Eizo, Hakusan, Japan) with a resolution of 1152 × 870 pixels, a refresh rate of 75 Hz, and an effective display size of 40.7 × 30.5 cm. Stimuli were viewed from a 67 cm distance. Stimulus presentation was controlled via the Psychtoolbox (Brainard, 1997; Kleiner et al., 2007) in MATLAB (The MathWorks, Natick, MA). Eye position of the right eye was recorded at 1000 Hz using the EyeLink 1000 (SR Research, Mississauga, ON, Canada) and the EyeLink Toolbox (Cornelissen et al., 2002). All stimuli were presented on a uniform gray monitor background.

#### Image database: Salient objects on structured backgrounds

**Figure 1.**
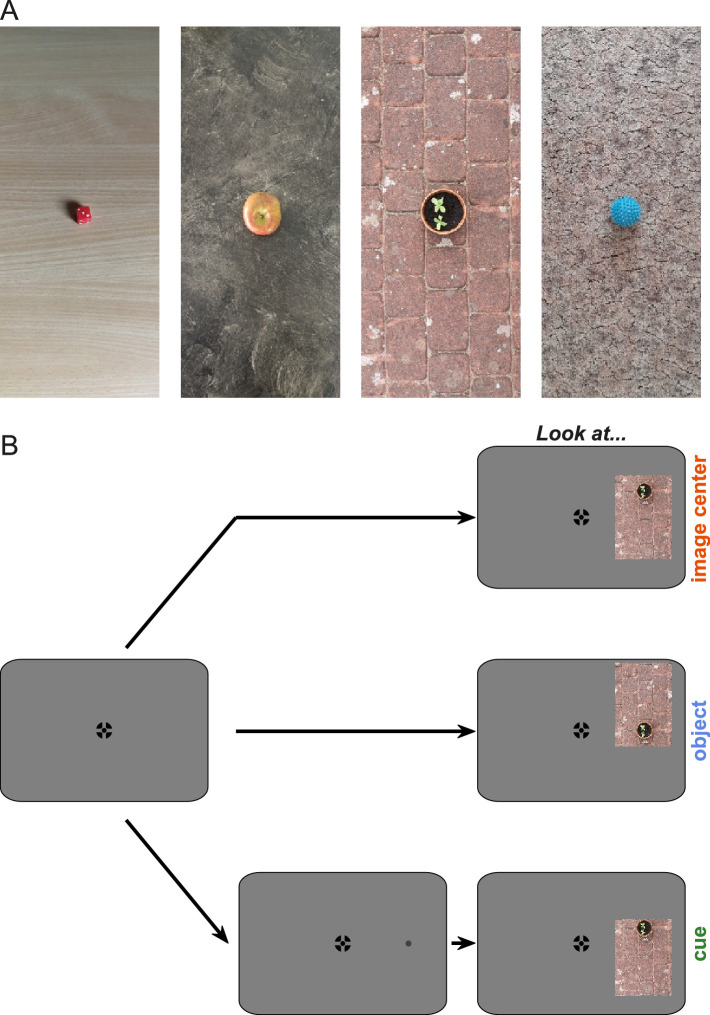
Experiment 1: Stimulus material and trial procedure. (A) Four example images from the 25 images used in Experiment 1. Images had a size of 270 × 630 pixels. When presented during the experiment, either the top or bottom part was cropped to displace the object relative to the image center. (B) Trial procedure. Participants started each trial by pressing a button on a keyboard while simultaneously looking at a central fixation cross (left). In each trial one image could appear left or right from fixation. In different conditions (recorded in different blocks) participants were instructed to either look at the center of the image, at the object, or at a location in between the image center and the object center that was cued by means of a small gray dot that appeared 120 ms before the image appeared. It was made explicit to participants that in all conditions the task would go along with a horizontal saccade. The font colors on the right-hand side denote the colors used for plotting the results of the respective conditions.

We took 43 photographs of salient objects of which 25 were selected for the experiment. All photographs show one object on a structured background (Figure 1A). Three people independently rated the suitability of all images for the purpose of the experiment on a scale from 1 to 10. Images were then selected based on the highest average ratings. Ratings were based on three criteria: (i) objects are placed in an environment in which they can normally be found, (ii) objects can easily be recognized, and (iii) objects stand out from the background with no further salient highlights in the background. The last criterion was assessed by visually inspecting saliency maps created with the Saliency Toolbox (Walther & Koch, 2006). Moreover, we made sure that objects were comparable in size. In a next step, images were cropped to a size of 270 (width) × 630 (height) pixels (aspect ratio of 9:21) with the object placed in the image center. Objects on average covered *M* = 1.67° horizontally (*SD* = 0.41°) and *M* = 1.91° vertically (*SD* = 0.59°) of visual space. All selected images are freely available from Zenodo (doi:10.5281/zenodo.5115492).

For the different conditions of the experiment, images were vertically cropped by 150 pixels (for the conditions *look at image center* & *look at object*) or 272 pixels (for the condition *look at cue*), resulting in an image size of 270 × 480 pixels (*look at image center* & *look at object*) or 270 × 358 pixels (*look at cue*), respectively. These were the images actually shown during the experiment. The reason for this cropping was to achieve a vertical displacement between image center and object center. Vertical displacements between image center and object center were 2.2° (*look at image center* & *look at object*) and 3.99° (*look at cue*). Objects were shifted to the upper half of the image by cropping the upper 150 or 272 pixel rows, and they were shifted to the lower part by cropping the lower 150 or 272 pixel rows. In half of the trials the images were horizontally mirrored to account for any possible left-right imbalances in the photographs.

#### Procedure

The experimental procedure is depicted in Figure 1B. As a fixation cross, we used a combination of bull's eye and hair cross (Thaler, Schütz et al., 2013) with an outer diameter of 0.5°. Participants could start a trial by looking at the central black fixation cross and simultaneously pressing the space bar on a keyboard. Either the image (conditions *look at image center* & *look at object*) or a small gray dot that served as a cue (condition *look at cue*) appeared at an eccentricity of 12° (left or right) after a uniform random interval between 500–1000 ms. In the *look at cue* condition, the cue was shown for 120 ms after which the image appeared. In each condition the image was shown for additional 300 ms after its foveation.

In the *look at image center* condition, participants were instructed to make a saccade to the center of the image as soon as it appeared. Like in the other two conditions, we explicitly told participants that this could be achieved by making purely horizontal saccades. The image appeared 12° left or right from the screen center and was vertically centered. In the *look at object* condition, images were additionally vertically shifted by 2.2° such that the object was always vertically centered on the screen. Participants were instructed to look at the center of the object and were again told that this could be achieved by making purely horizontal saccades. In the *look at cue* condition, participants were instructed to look at the location of the cue as soon as the cue was replaced by the image. Again, participants were explicitly told that this could be achieved by making purely horizontal saccades.

Each of the three conditions was recorded in a different block. Each block contained 200 trials: 25 images × two saccade directions (left vs. right) × two vertical displacements (up vs. down) × two horizontal versions (mirrored vs. original). Thus, each image was shown eight times in each condition. We balanced the order of conditions across participants as well as the trial order within a block. All three conditions were recorded within one session of approximately one hour with breaks in between blocks. At the beginning of every block the eye tracker was calibrated using a nine-point grid procedure.

#### Data analysis

We measured eye movements of the right eye with a sampling rate of 1000 Hz. Onsets and offsets of saccades were defined offline using the EyeLink 1000 algorithm, which uses a combination of velocity (30°/s), acceleration (8000°/s^2^) and displacement (0.15°) threshold. The temporal difference from image onset to saccade onset was taken as saccade latency (*look at image center* & *look at object*) and Δ*t* (*look at cue*), respectively. We referenced vertical gaze position relative to the screen midline and recoded the data such that the displacement of the object (*look at image center* & *look at cue*) or the image center (*look at object*) goes along with positive values. Recoding also accounted for any hypothetical vertical biases in the data (e.g., if participants preferred to look at the upper border of objects) and any (tiny) vertical imbalances in the photographs.

To analyze endpoints over time, we used a cluster-based permutation approach (SMART, smoothing method for the analysis of response time courses, van Leeuwen et al., 2019) where the data are first temporally smoothed for every individual, then a weighted time series is constructed that considers the data distribution of every individual and, finally, a cluster-based permutation test is performed. This analysis procedure including all parameters was equivalent to Wolf and Lappe (2020). Thus, data were smoothed using a Gaussian kernel of 16 ms width at a 1 ms resolution, and we used 10,000 permutations for every test. For every comparison we report four values: the *p* value, the cluster strength of the nonpermuted data (*t*), the 95^th^ percentile of the permuted distribution, and the time window of the significant cluster. The 95^th^ percentile of the permuted distribution is the critical *t* value (*t_crit_*) to which the cluster strength of the nonpermuted data is compared. The *p* value is given by the relative position (i.e., percentile) of the nonpermuted cluster strength in the distribution of all permuted cluster strengths.

We evaluated time courses of saccade endpoints in a time window between 50–300 ms (*look at image center* & *look at object*) and -50 to 300 ms (*look at cue*). These time windows covered 95.0 % (*look at image center*), 98.2% (*look at object*), and 97.7% of trials (*look at cue*), respectively (Figure 4, top row). Moreover, we discarded trials with a horizontal amplitude below 6° (82 trials, < 0.8%) and a vertical saccade endpoint that deviated more than 4° from the monitor midline (two trials).

Any deviation in the mean saccade endpoint could arise because either the whole endpoint distribution is biased or because gaze was captured by the salient object/image center in a fraction of trials. In the former case, the endpoint distribution should have a unimodal profile, whereas, in the latter case, the distribution of endpoints should have a bimodal profile. To reveal whether the object and the image center biased the distribution of endpoints or captured endpoints in a fraction of trials, we divided vertical endpoints in 20 equally sized bins between −2.5° and +2.5° and fitted two models to this data: a scaled Gaussian (single-Gaussian model) and the combination of two scaled Gaussians (dual-Gaussian model). The single-Gaussian model had three free parameters: the mean of the Gaussian, its standard deviation, and a scaling parameter that scaled the Gaussian up or down by means of multiplication. The dual-Gaussian model had five free parameters, the two means, the two scaling parameters, and the standard deviation. The standard deviation was assumed to be identical for both Gaussians because it is thought to reflect an individual's oculomotor variability, which is supposed to be the same no matter which target is selected. Yet, conclusions did not change when the standard deviation was allowed to differ between the two Gaussians. Model fits were first evaluated by the Bayesian information criterion (BIC), which also takes the number of free parameters into account and subsequently compared using information weights (Burnham & Anderson, 2002). Information weights add up to 1, range from 0 to 1, and higher values denote a higher evidence for a particular model. We fitted each of the two models to the data of every individual in every condition and only considered trials with reaction times in the time window revealed by the SMART analysis (see Results). For every condition, information weights for the two models were compared using Wilcoxon signed-rank tests, because information weights were not normally distributed.

### Results

**Figure 2.**
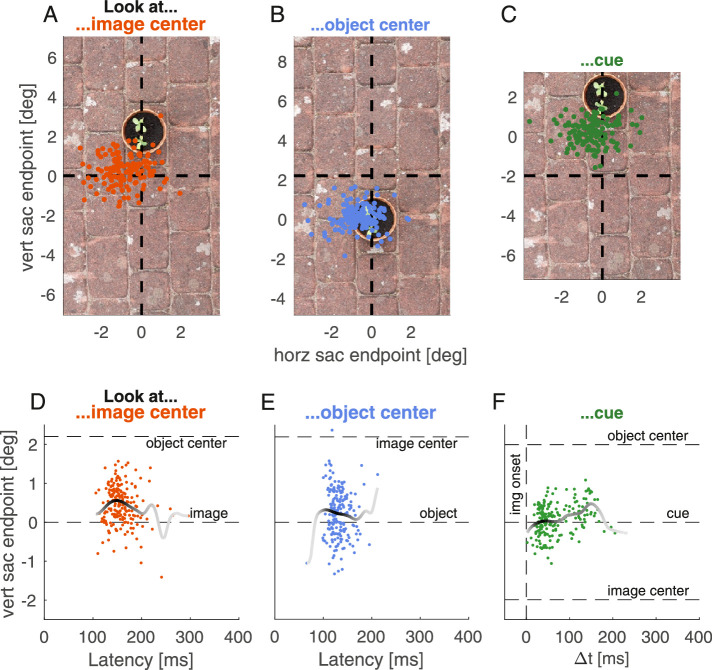
Experiment 1: Distribution of individual endpoints in space and time. (A–C): Vertical and horizontal saccade endpoints of all participants for two example images in the *look at image center* (A), *look at object* (B), and *look at cue* condition (C). The intersection of dashed lines denotes the image center. Location (0,0) is the instructed location. Each data point is the saccade endpoint of one trial and each panel contains up to eight trials per individual. Horizontal saccade direction was recoded to correspond to rightward saccades. Thus, the location of the fixation cross was (-12,0) and horizontal endpoints below 0° correspond to saccadic undershoot. (D–F): Endpoint time courses of one example participant. Vertical saccade endpoints as a function of saccadic reaction time for all three conditions. Dashed horizontal lines indicate the location of image center and object center, respectively (and cue in F). Data points are endpoints of individual trials. The solid line represents a weighted average that was computed by means of a sliding Gaussian window with a standard deviation of 16 ms. The darker the line the more data points contribute to the estimate of that time point.

In Experiment 1 a salient object and the center of the image competed for visual selection. The three conditions differed in terms of the vertical position of the object and image center as well as the instructions provided to participants. In condition 1 participants were instructed to look at the center of an appearing image when the object was vertically displaced (*look at image center*). Conversely, in condition 2 participants were instructed to look at the object while the image center was vertically displaced (*look at object*). In condition 3 participants were instructed to saccade to a cued location that was vertically in between the image center and object (*look at cue*). Figure 2A–C shows saccade endpoints of all participants in all three conditions on an example image. We analyzed saccade endpoints as a function of saccadic reaction time and reconstructed individual time courses. Figure 2D–F shows vertical endpoints as a function of saccadic reaction time for one example participant.

**Figure 3.**
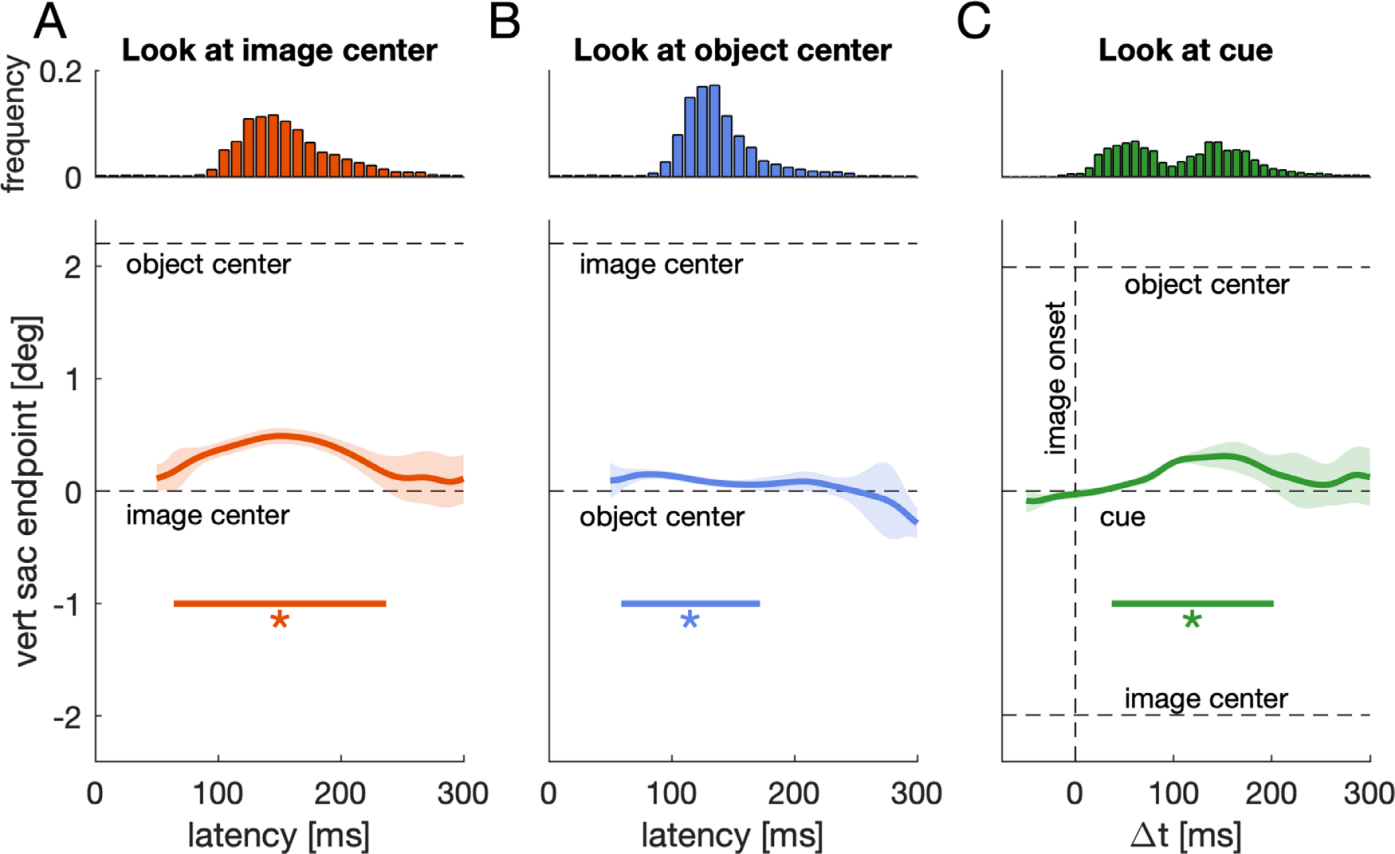
Experiment 1: Main results. Vertical saccade endpoints (lower panels) for all three conditions of Experiment 1 and reaction time histograms (upper panels) for the respective conditions in the panel below. Dashed horizontal lines indicate the location of image center and object center, respectively. Solid horizontal lines and asterisks indicate a significant cluster in the respective time window. Shaded regions denote 95% confidence intervals. Confidence intervals result from one-sample testing (van Leeuwen et al., 2019) against baseline (0°). Any significant cluster thus shows a bias away from the instructed location. (A) Condition 1: Look at image center. Endpoints relative to the image center as a function of saccade latency. (B) Condition 2: Look at object center. Endpoints relative to the object center as a function of saccade latency. (C) Condition 3: Look at the cued location. Endpoints relative to the cued location as a function of the temporal difference between image onset and saccade onset.

Figure 3A shows the reconstructed time course of vertical endpoints aggregated over all participants when participants were instructed to look at the image center. Endpoints were significantly biased toward the salient object, *p* < 0.001, *t* = 1756, *t_crit_* = 138.8, in the time window from 64–237 ms. In condition 2, when participants were instructed to look at the center of the object and the image center was vertically displaced, endpoints were systematically biased toward the image center and away from the salient object for saccades initiated in a time window between 59–172 ms after image onset (Figure 3B), *p* < 0.001, *t* = 589.9, *t_crit_* = 162.3. A comparison between the two time courses (i.e., Figure 3A vs. 3B) revealed that the bias toward the salient object was stronger than the bias toward the image center, *p* < 0.001, *t* = 1232, *t_crit_* = 243.4, 79–225 ms. We found no evidence that these time courses changed with repeated exposure to the images (Supplementary Fig. 1).

The third condition aimed to reveal which of the two biases, saliency bias or the bias exerted by the image center, dominates if they exert a pull in different directions. When asked to look at a cued location in between salient object and image center, saccade endpoints were biased toward the salient object, *p* < 0.001, *t* = 1283, *t_crit_* = 140.8, in the time window from 79–225 ms.

We next asked whether image center and salient objects biased saccade endpoints or whether they rather captured endpoints. Salient objects in proximity of the target are said to bias endpoints continuously due to averaging responses and center-of-gravity computations in the priority map (for review, see van der Stigchel & Nijboer, 2011). A priority map is a hypothetical retinotopic representation of space that combines bottom-up and top-down information and codes potential saccade targets by peaks of activity. Characteristics of such a map can be found in several sites along the oculomotor circuitry (for review, see Bisley & Mirpour, 2019). Typically, the highest peak is selected as the next saccade target. Yet, a bias can occur, especially when two peaks (two potential targets) are in spatial proximity, in which case the saccade endpoint is determined by performing a weighted average of the two peaks. In contrast to this weighted average, salient objects have also been shown to capture gaze and attention (e.g., Theeuwes et al., 1998), thereby emphasizing a rather dichotomic mechanism of oculomotor and attentional selection. If, for example, the salient object captured gaze in the *look at image center* condition, then a saccade would be erroneously directed toward the salient object. If such a capture occurred in half of the trials, the mean vertical saccade endpoints would be in between the salient object and the image center. The same mean vertical endpoint would be expected if all endpoints were biased to this location in between image center and object. Therefore, the aggregated time courses (Figure 3) do not allow to distinguish between these two alternatives since they could be explained either by a fraction of trials in the respective time window being captured by the salient object or by a continuous shift by the entire endpoint distribution to an intermediate location. However, the two cases can be distinguished by looking at the distribution of endpoints. In case of oculomotor capture, the distribution of vertical endpoints should exhibit a bimodal profile with one peak centered at 0° and a second peak centered close to +2°. In case of center-of-gravity computations (a continuous bias), the distribution of vertical endpoints should exhibit a unimodal profile with a peak in between the two locations.

**Figure 4.**
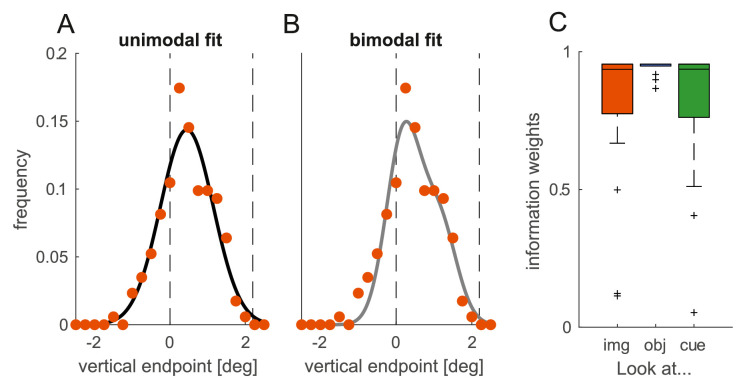
Experiment 1: Endpoints are biased rather than captured. (A, B): Vertical endpoint distribution (orange data points) from an example participant in the *look at image center* condition together with a unimodal (black line in A) and a bimodal model fit (gray line in B). Dashed vertical lines indicate the location of the image center and salient object. (C) Distribution of information weights for the unimodal model (Burnham & Anderson, 2002). Information weights for both model fits add up to and higher values in the figure denote higher evidence for the unimodal model.

To distinguish between these two alternatives, we tested whether the distribution of endpoints can be better explained by a unimodal or a bimodal distribution (Figures 4A & B). We fitted two models to the vertical endpoint distribution to the individual data of each condition: a Gaussian as well as the combination of two Gaussians. Importantly, we only considered trials within the identified clusters (Figure 3). We compared the two model fits by computing information weights (Burnham & Anderson, 2002; Figure 4C) derived from the Bayesian Information Criterion (BIC). A higher information weight indicates a better model fit. We found that endpoint distributions of all three conditions were better explained by a unimodal distribution as indicated by higher information weights for the unimodal model: For condition 1 (*look at image center*), information weights for the unimodal model were higher for 15 out of 18 participants, *Z* = 3.29, *p* = 0.001. For condition 2 (*look at object*) this was true for all 18 participants, *Z* = 3.72, *p* < 0.001. For the third condition (*look at cue*) information weights for the unimodal model were higher for 16 out of 18 participants, *Z* = 3.2, *p* = 0.0014. This shows that salient objects and the image center biased the entire endpoint distribution within a certain time window rather than a fraction of trials in that time window being captured by the salient object or image center.

### Discussion of experiment 1

We measured eye movements to images containing one salient object displaced from the image center. In different blocks we asked participants to either look at the image center (condition 1), the object center (condition 2), or at a cued position halfway in between the two (condition 3). We found that vertical endpoints were biased toward the salient object in the *look at image center* condition (Figure 3A) and to the image center in the *look at object* condition (Figure 3B) depending on saccade latency. The bias toward salient objects dominated if both biases were effective in different directions (Figure 3C; *look at cue*).

Our results thus replicate that salient stimuli bias saccades within a certain time window after target appearance, resulting in a center-of-gravity response (e.g., Ottes et al., 1985; Coëffé & O'Regan, 1987; Wolf & Lappe, 2020). Moreover, the results showed the existence of a similar automatic scene-dependent center bias for early saccadic responses (Figure 4B), independent of any image features and salient items in the image. This is further evidence that the center bias depends on time and can be reduced by delaying the initial response to the image (Rothkegel et al., 2017). It additionally shows that early central fixation tendencies do not only occur because the image center constitutes a strategically advantageous location for image exploration, but that gaze can be automatically biased toward the image center when looking at an image. Whereas the observed bias toward the image center is comparatively small, it has to be noted that this bias was measured as the deviation away from (i) the instructed endpoint, (ii) the center of the salient object, (iii) the screen midline as well as (iv) the vertical starting position of the saccade. Whereas all these aspects enable attributing the observed bias to the vertical image location and thus the location of the image center, they most likely attenuate the automatic bias toward the image center. For example, if images were positioned in the center of the monitor, this bias might have been substantially stronger because of the additional bias toward the screen center (Bindemann, 2010).

Both biases were not maximal for the earliest responses but took time to unfold. This becomes especially apparent when participants could plan and execute a saccade before the actual appearance of the saccade target and saccadic endpoints can be analyzed over a broader range of reaction times (Figure 3C; Wolf & Lappe, 2020). Whereas the bias toward salient objects was strongest for responses in the middle of the saccade latency distribution (Figure 3A), the bias toward the image center was strongest for the earlier half of the responses (Figure 3B). In contrast to condition 1 and 2, the response distribution in condition 3 showed a bimodal profile. The later peak in reaction times overlapped with the peak endpoint bias toward the salient target, whereas the earlier peak in reaction times most likely reflects anticipatory saccades and thus saccades to the cue rather than toward the image. The bimodality in the distribution of reaction times can most likely be attributed to saccadic inhibition (Walker et al., 1997; Reingold & Stampe, 1999; 2002; Buonocore & McIntosh, 2008), a decline in saccade frequency, approximately 100 ms after large changes in the visual scene. Theoretically, this bimodal profile might also be a consequence of combining within-participant and between-participant variability. However, the dip in saccade frequency around 100 ms after image onset can also be observed on the individual level (Figure 2F), suggesting that it can be attributed to saccadic inhibition.

## Experiment 2

Where does the automatic bias toward the image center come from? The existence of an image-dependent center bias shows that the oculomotor system must have access to the image outline and that this information is used for saccade programming—resulting in the observed bias toward the center of gravity (e.g., Kowler & Blaser, 1995; Vishwanath & Kowler, 2003; Bindemann et al., 2009). This suggests, furthermore, that at the onset of the image on the monitor the visual system determines the boundaries of the image before making the saccade. This process is similar to figure-ground segregation, the process of telling apart a figure from the background (Lamme, 1995; Roelfsema et al., 2002), only that in our setup the figure (the image) needs to be segregated from the monitor background.

Figure-ground segregation is thought to rely on distinguishable subprocesses (feature extraction, boundary detection, region filling) that have been shown to operate at different time courses (Romani et al., 1999; Heinen et al., 2005; Poort et al., 2012; Self et al., 2013). Within different areas and layers of the visual cortex, the visual response to the onset of a figure-ground stimulus can start as early as below 50 ms after stimulus onset, with detection of the figure-background boundary starting approximately 60–70 ms, and region filling of the figure approximately 100 ms after stimulus onset (Poort et al., 2012; Self et al., 2013). In our experiments, although information about the image boundary would have to be passed on to the oculomotor network first, the time windows for the endpoint biases by the object in the image or by the image itself overlap with the time window of figure-ground segregation processes. Yet, these timings will likely be affected by differences in stimuli (e.g., strong luminance transients) or differences in data analysis. For example, it has to be considered that our time course of endpoints is smoothed and might be a low-pass filtered version of the actual underlying time course.

**Figure 5.**
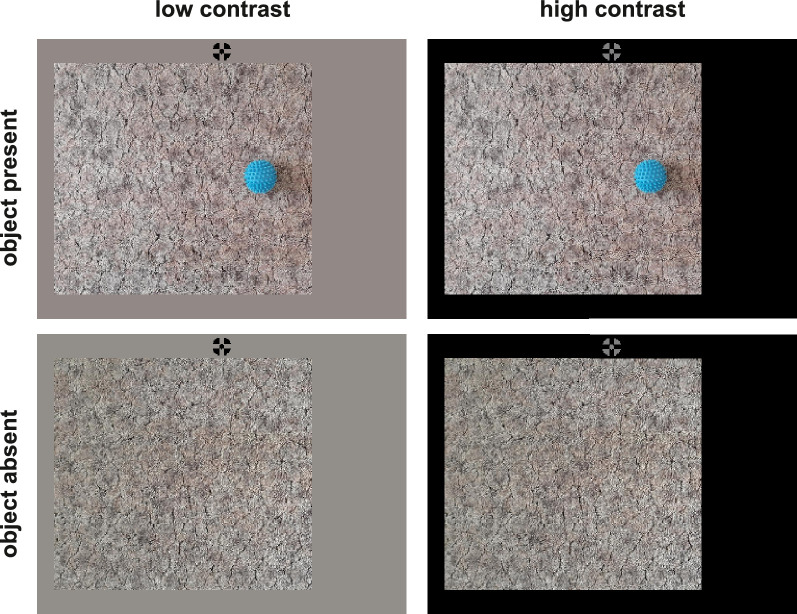
Experiment 2: Stimulus manipulation and experimental design. Participants were instructed to visually explore images for two seconds and were told that they have to answer questions about the images afterward. Images were structured backgrounds, either containing an object (top row) or containing no object (bottom row). Shown images had a size of 800 × 720 pixels. The monitor background was either set to the average RGB value of the image background (low contrast; left column) or it was set to black to maximize the luminance contrast between image and monitor background (high contrast; right column). Participants started each trial by looking at a vertically displaced fixation cross that was horizontally positioned in between image center and the (potential) object location. The fixation cross disappeared before the image appeared (gap paradigm) and is only depicted in the figure for illustrative purposes.

If the center bias relies on figure-ground segmentation processes, we would expect that it should be particularly pronounced when the image clearly stands out from the monitor background (i.e., high contrast) and that it should be attenuated when it is difficult to tell apart the image and the monitor background (i.e., low contrast). The first purpose of Experiment 2 was to test this hypothesis. We therefore manipulated the luminance contrast between monitor and image background, which could either be high or low. This was done to make the image itself either salient with respect to the background, or not. The second purpose was to replicate the dominance of salient objects over the center bias with an experimental approach that is more established to measure central fixation behavior. Thus, to put this conclusion on more solid ground, we asked a new set of participants to freely explore larger landscape-oriented versions of our images for an extended duration. This allowed us to analyze the subsequent gaze position while exploring these images in addition to the analysis of initial endpoints. To reveal whether salient objects overwrite central fixation behavior during free exploration, we manipulated the presence of objects. Images either contained one salient object on a structured background (object present condition; identical to Experiment 1) or only showed the structured background without the object (object absent condition; Figure 5). Equivalent to Experiment 1, object and image center were horizontally displaced by cropping either the right-hand side or the left-hand side of the image. If salient objects prevail over the center bias, then we would expect that initial saccade endpoints and the subsequent gaze position are less biased toward the image center in the object present compared to the object absent condition. If the bias toward the image center is affected by figure-ground segregation, we would expect that the initial saccade endpoint and the subsequent gaze position should be more strongly biased toward the image center in the high contrast condition. This should be particularly pronounced in trials without a salient object.

### Methods

#### Participants

We recorded data of 24 individuals who had not participated in Experiment 1 (18 female, six male, median age: 21 years, age range: 17–48 years). All participants had normal or corrected-to-normal vision and were naïve with regard to the purpose of the experiment. Participants were undergraduate students from the University of Muenster and received a reimbursement of 8€/hour or course credit for participation.

#### Stimuli and design

Stimuli were 32 images. Half of them belonged to the images that were used for Experiment 1. The other 16 images were photographs of the same background but without the salient object. Thus, we had 16 pairs of identical backgrounds, once with and once without a salient object. All images used in Experiment 2 are available from Zenodo (doi:10.5281/zenodo.5115492).

Images were edited to have a size of 1280 (width) × 720 (height) pixels with the object centered in the middle. During the experiment, a subregion of the images (800 × 720 pixels) was selected in each trial by cropping either the right-hand or the left-hand 480 pixels, causing a displacement of 240 pixels (7.04°) between the object and image center. The images shown during the experiment thus covered approximately 23.5° × 21.1° of the visual field.

In half of the trials the monitor background was set to black at the beginning of a trial to create a high contrast between the image background and monitor background (high contrast condition; Figure 5, right column). In the other half, the monitor background was set to an RGB value that caused a low contrast between the image background and monitor background (low contrast condition; Figure 5, left column). These RGB values were determined by averaging RGB values across the whole image (1280 × 720) but sparing out the central 100 × 100 pixels (the region where the object was or could be) so that the RGB value reflects the image background and would not be biased toward the object for object present images. The design thus comprised the two factors object (present vs. absent) and image-monitor contrast (low contrast vs. high contrast).

#### Procedure

Participants were instructed to carefully inspect each image as they would have to answer questions about the images afterward. At the end of the experiment, participants were debriefed that there were no questions, and this instruction was chosen to assure they would thoroughly explore the images.

Participants started each trial by looking at a vertically displaced fixation cross. Depending on the contrast condition, the fixation cross was either black or medium gray (Figure 5). The fixation cross was horizontally centered on the screen but vertically displaced (up or down) by 11°. After a random interval the fixation cross was removed from the screen and the image appeared 120 ms later (gap paradigm). To minimize the number of anticipatory saccades toward, for example, the screen center before the appearance of the image, the image would only appear if the distance between the current gaze position and the fixation cross (while it was still displayed) was less than 2.5°. The image was vertically centered but shifted to the left or right by 3.52° (120 pixels). In object present trials, object center and image center were horizontally separated by 7.04° with the screen center halfway in between the two. Thus, the overall eccentricity of object and image center relative to the initial fixation position was 11.55°. Images were presented for two seconds.

The experiment comprised 512 trials: 16 image backgrounds × two object status (present vs. absence) × two monitor backgrounds (low contrast vs. high contrast) × two image shifts (left vs. right) × two fixation cross positions (top vs. bottom) × two horizontal versions (mirrored vs. original). Trials from different conditions were randomly interleaved and all trials were recorded within one session of approximately 40 minutes with two breaks in between. The eye tracker was calibrated at the beginning of the session and after every break.

#### Data analysis

The analysis of horizontal endpoints over time was equivalent to the analysis of vertical endpoints in Experiment 1, using the SMART Toolbox (van Leeuwen et al., 2019) again. Additionally, we analyzed horizontal endpoints of secondary saccades as a function of the temporal difference between the onset of the second saccade and the onset of the image (Δt). For the analysis of these secondary saccades, we used a Gaussian kernel of 32 ms (instead of 16 ms), because the data was more widely distributed than the latency of primary saccades. We evaluated time courses of saccade endpoints in a time window between 40–250 ms (primary saccades) and 150–500 ms (secondary saccades). These time windows covered 91.9% (primary) and 81.6% (secondary) of trials.

We compared saccade latencies of primary saccades and the fixation duration in between primary and secondary saccades using a 2 × 2 repeated-measured ANOVA with the two factors object (present vs. absent) and image-monitor contrast (low vs. high). For the analysis of primary saccades, we did not consider saccades with latencies below 80 ms, which were classified as anticipatory saccades. The frequency of anticipatory saccades was compared using Wilcoxon signed-rank tests.

**Figure 6.**
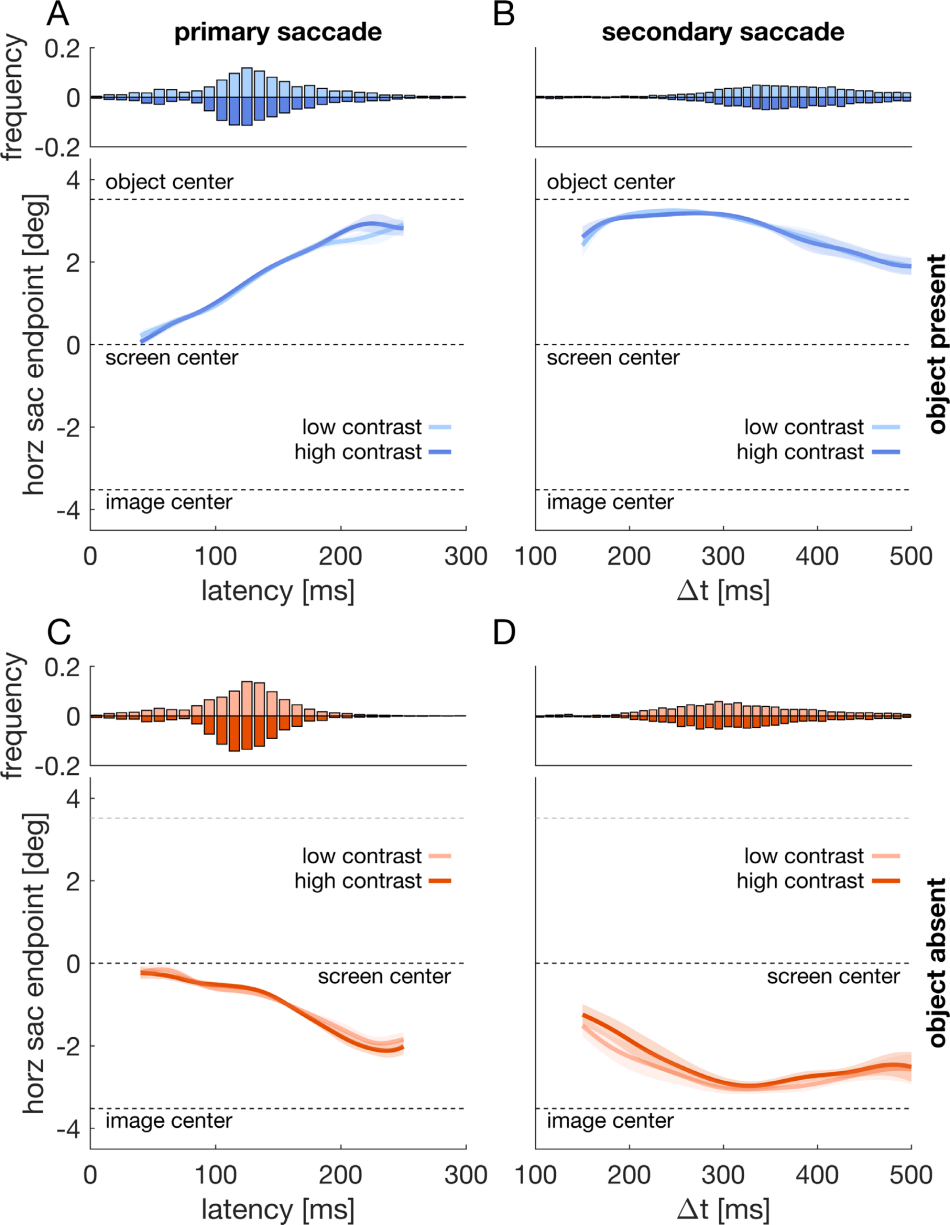
Experiment 2: Initial orienting toward images. Horizontal saccade endpoints over time for primary saccades (A, C) and for secondary saccades (B, D) to images containing an object (top row; A, B) or images containing no object (bottom row; C, D). Shaded regions are 95% confidence intervals that result from comparing the two depicted time courses against each other (van Leeuwen et al., 2019). The top of each panel shows reaction time histograms for the respective conditions plotted below. The bin size of all histograms is 10 ms.

Furthermore, we evaluated the Euclidian distance between gaze and image center (e.g., Rothkegel et al., 2017) over time. We compared distance time courses with a cluster-based permutation approach (Maris & Oostenveld, 2007) using custom scripts in MATLAB. The analysis of gaze position, unlike the analysis of endpoints, does not require smoothing and reconstructing a weighted time series, because there is data for all time points. Consistent with the other analyses, we evaluated these gaze time courses using 10,000 permutations at a 1 ms resolution. To evaluate whether differences in the Euclidian distance between gaze and image center are due to a bias in mean saccade endpoints or due to changes in endpoint variability, we compared mean horizonal endpoints, mean vertical endpoints as well as horizontal and vertical endpoint variability for the second to fifth saccade using 2 × 2 × 4 repeated-measures ANOVAs with the factors object, image-monitor contrast, and saccade number. To correct for multiple testing, *p* values of the four ANOVAs were compared to a corrected alpha level of 0.0125. Primary saccades were not included in this analysis because differences in mean endpoints can be attributed to differences in saccade latencies (see below; Figure 6).

### Results

In all conditions, endpoints of the earliest saccades were directed close to the screen center (Figures 6A & C). Especially, the very early saccades (e.g., latency of <50 ms) were most likely made in anticipation of an image. With increasing saccade latency, endpoints more strongly depended on the experimental conditions. In object present conditions, endpoints became directed closer to the object (Figure 6A). In the conditions without object, endpoints became instead directed closer to the image center (Figure 6B). We compared endpoints over time for object present and object absent trials, aggregated over the two image-monitor contrasts (thus, comparing the aggregated time course from 6A with the one from 6C). These time courses differed significantly, *t* = 3520, *t_crit_* = 108.7, *p* < 0.001, 50–250 ms. This was also true for secondary saccades (i.e., the second saccade after image onset), *t* = 8363, *t_crit_* = 207, *p* < 0.001, 150–500 ms. We compared low contrast and high contrast time courses in the object present and absent condition, respectively. In both conditions, object present and absent trials, endpoints of primary saccades did not differ for the two contrast conditions (Figures 6A & C). The same observation holds for the secondary saccades (Figures 6B & D).

The gap paradigm is known to produce anticipatory primary saccades, i.e., saccades with a latency smaller than 80 ms. In our sample this applied on average to 12.9% of trials. We observed a tendency for more anticipatory saccades for low compared to high contrast trials when the object was absent (*M* = 14.3% vs. *M* = 11.3%), *Z* = 1.98, *p* = 0.048, but not when the object was present (*M* = 13.9% vs. *M* = 12%), *Z* = 1.63, *p* = 0.104 (see histograms in Figure 6A & C). The horizontal endpoint of all anticipatory saccades was almost perfectly aligned to the screen center (*M* = 0.013°). For the remaining primary saccades, we observed shorter latencies when the object was absent compared to when it was present (*M* = 133.5 ms vs. *M* = 149.7 ms), *F*(1,23) = 89.7, *p* < 0.001, and when the image-monitor background was high compared to when it was low (*M* = 139.8 ms vs. *M* = 143.3 ms), *F*(1,23) = 6.11, *p* = 0.021 (see histograms in Figure 6A & C). For fixation durations in between primary and secondary saccades, we observed longer fixation durations when objects were present compared to when they were absent (*M* = 226.5 ms vs. *M* = 183.1 ms), *F*(1,23) = 28.8, *p* < 0.001, but we found no evidence that fixation durations were affected by the image-monitor contrast, *F*(1,23) = 1.28, *p* = 0.271 (low: *M* = 206.4 ms; high: *M* = 203.2 ms).

**Figure 7.**
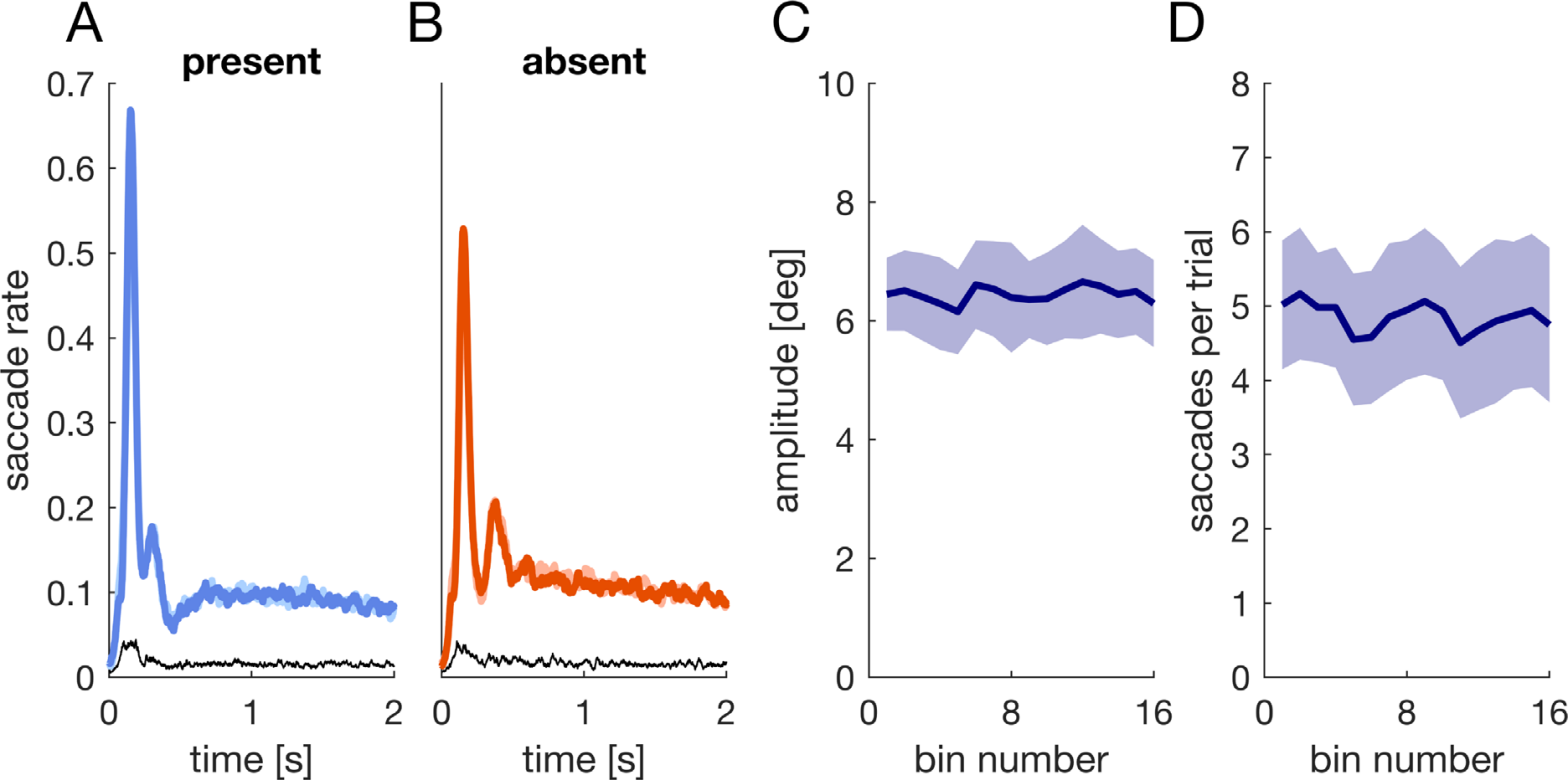
Experiment 2: Exploration behavior over time. (A, B) Average saccade rate for object present (A) and object absent trials (B), separately for high contrast (saturated colors) and low contrast trials (faint colors, mostly hidden). The thin black line denotes the 95% confidence interval of the difference between the two lines and is plotted separately to enhance visibility. (C, D) Mean amplitude (C) and mean number of saccades per trial (D) over the course of the experiment. Trials were binned in 16 bins of 32 trials each. Shaded regions are the 95% confidence intervals of between participant variability.

We additionally analyzed gaze position on the images after the initial orienting. Participants were instructed to carefully inspect each image and were told that they would have to answer questions about the images after the experiment. Yet, given that images only contained either one or no salient object, we worried that participants might have made only one or two saccades and then waited for the next trial. To obtain an estimate for exploration behavior, we computed the saccade rate over the trial duration (i.e., the fraction of trials with a saccadic sample at that time point). The saccade rate showed two peaks reflecting the synchronized primary and secondary saccades and subsequently reached an asymptote at a rate of around 0.1, which approximately corresponds to three 30–40 ms saccades per second (Figure 7A–B), i.e., a normal rate for visual inspection of images. Across the whole trial duration, the average saccade rate was *M* = 0.113 for object present and *M* = 0.126 for object absent trials and similar for the two image-monitor contrasts.

Each image was shown multiple times during the experiment to balance experimental factors (see Procedure). Thus, exploration behavior might have changed over the course of the experiment as participants were repeatedly exposed to the same images. To assess whether the quality of exploration behavior changed over the course of the experiment, we computed the mean amplitude and the mean number of saccades per trial (not including saccades with an amplitude < 1° to not include microsaccades). Both, the mean amplitude (*M* = 6.4°) and the mean number of saccades per trial (*M* = 4.9) did not change across the experiment (Figures 7C–D).

**Figure 8.**
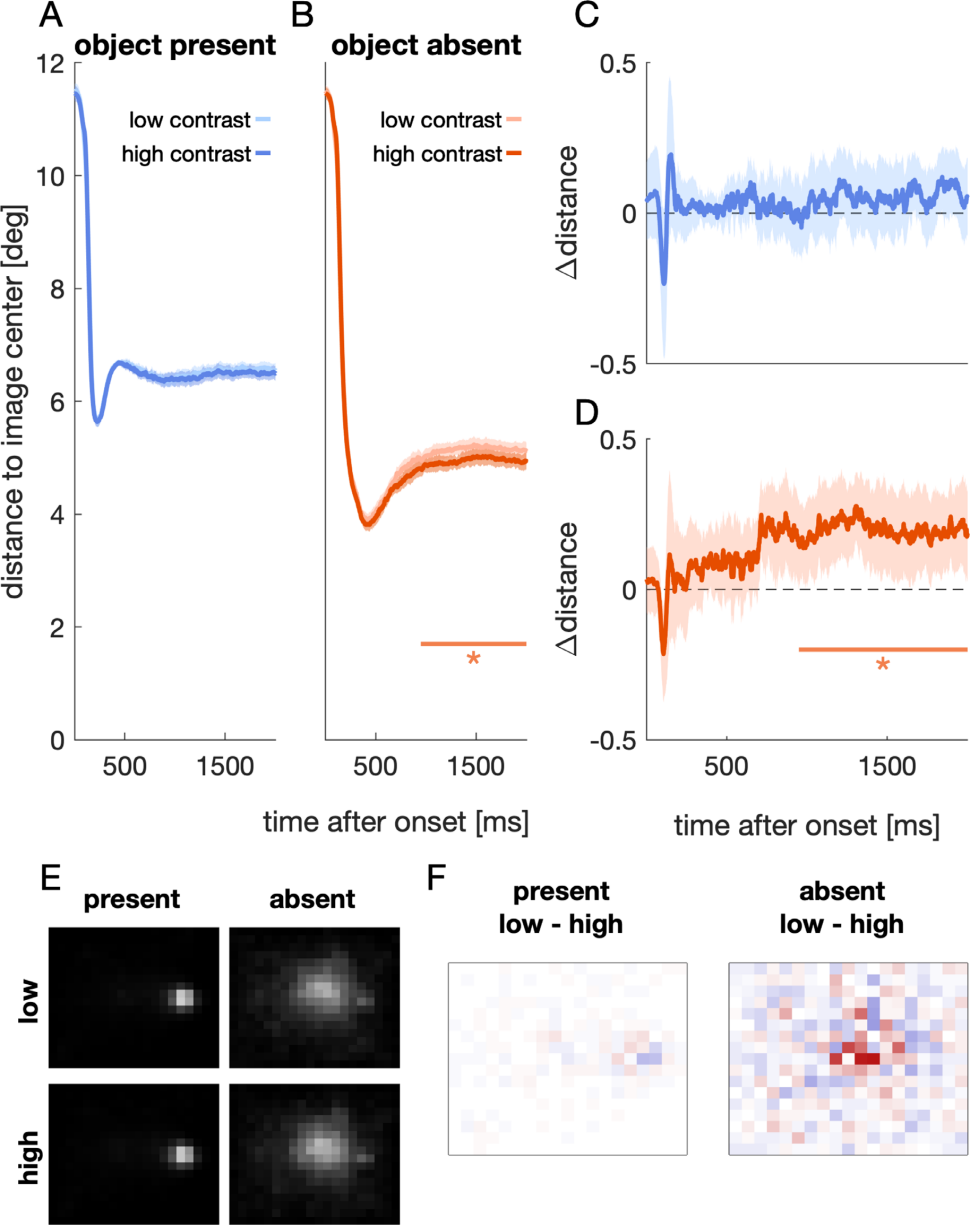
Experiment 2: Temporal and spatial distribution of gaze. (A, B): Euclidian distance to image center over time when objects were present (A) or absent (B). Horizontal lines and asterisks denote a significant difference between the two conditions. (C, D): Differences between the two time courses shown in (A) and (B). Positive values indicate that gaze was closer to the image center in the high contrast condition. Shaded regions are 95% confidence intervals. (E): Relative probability of image regions being looked at for each of the four respective conditions. Images were divided into bins with a size of 40 × 40 pixels. Brighter bins denote a high fraction of time points that gaze was detected within that bin. (F) Differences for the corresponding low and high contrast conditions depicted in (E). Blue values denote higher values for the low contrast condition and red values denote higher values for the high contrast condition.

Figure 8E shows the spatial distribution of gaze position between offset of the primary saccade and the end of a trial. Whereas most fixations were on or near the object in object present trials (Figure 8E, left panels; Figure 5), gaze was more widely distributed with a peak in the image center when no object was present (Figure 8E, right panels). However, gaze was not symmetrically distributed across the image center but closer to the screen center and initial fixation cross (Supplementary Fig. 2). In Figure 8E this corresponds to the right half of the object absent panels. Figure 8F shows the difference between the low and high contrast for object present and absent trials, respectively. Differences between low and high contrast were more pronounced for object absent trials. Particularly the image center was more likely to be looked at with a high contrast between image and monitor background.

Figure 8A–D shows the distance to image center over time (e.g., Rothkegel et al., 2017) for object present (Figures 8A & C) and object absent trials (Figures 8B & D), respectively. The time courses for object present versus absent trials differed, both for the high contrast, *t* = 18734, *t_crit_* = 489.7, *p* < 0.001, time window: 208–2000 ms, and the low contrast condition, *t* = 16653, *t_crit_* = 493.2, *p* < 0.001, time window: 207–2000 ms. In object absent trials, the distance to image center was decreased for the high contrast compared to the low contrast condition, *t* = 3163, *t_crit_* = 490.6, *p* < 0.001, time window: 949–2000 ms. In sum, a bias toward the image center was only found in object absent trials (Figure 8E), and this bias was further modulated by the contrast between image background and monitor background (Figures 8D & F).

**Figure 9.**
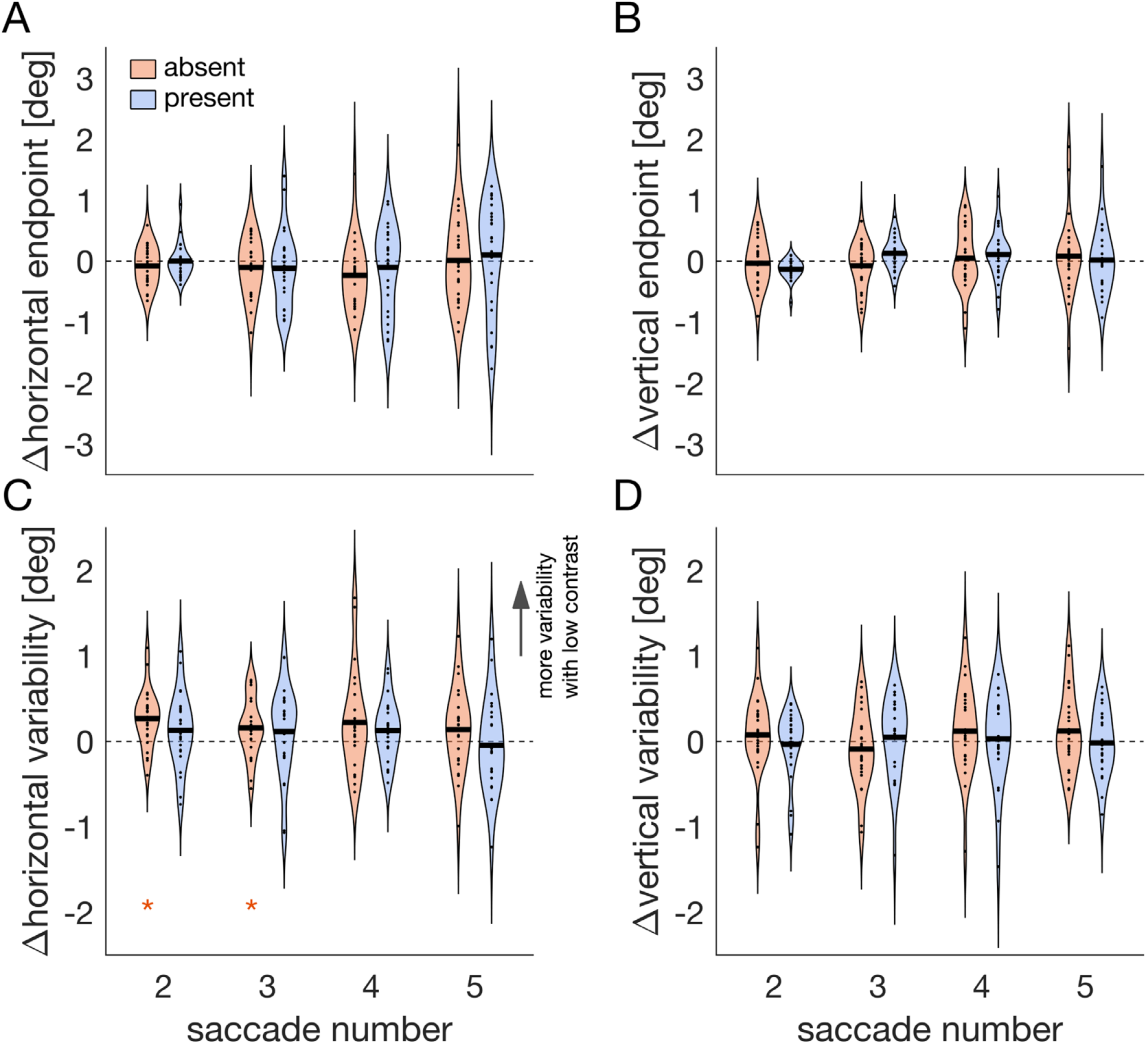
Experiment 2: Image-monitor contrast affects horizontal endpoint variability. Violin plots of the difference between the low and high image-monitor contrast condition for saccades two to five within a trial, separately for the object absent (orange) and object present condition (blue). Black solid lines denote the mean across participants and black data points are the mean difference of each individual. Asterisks mark a significant post hoc comparison (*p* < 0.05). Difference in (A) mean horizontal endpoint, (B) mean vertical endpoint, (C) horizontal endpoint variability, and (D) vertical endpoint variability.

There are two possible explanations of how the image-monitor contrast might have affected the distance between gaze and image center. First, participants may have selected, on average, a location farther away from the image center. This should be reflected in a bias in the mean saccade endpoints. Second, endpoint selection may have been less consistent. This would be reflected in the individual endpoint variability. Especially if the average gaze position is close to the screen center, a higher endpoint variability will increase the average distance to the image center (Figure 8D). To distinguish between these two possibilities, we analyzed the mean horizontal endpoint, the mean vertical endpoint as well as the mean individual horizontal and vertical endpoint variability, respectively. Figure 9 shows the difference between the low and high contrast condition of these four metrics, for the second, third, fourth, and fifth saccade, respectively.

We found no evidence that the image-monitor contrast affected the mean horizontal endpoint, *F*(1,23) = 1.32, *p* = 0.26, the mean vertical endpoint, *F*(1,23) = 0.27, *p* = 0.609, or the vertical endpoint variability *F*(1,23) = 0.61, *p* = 0.443 (main effects contrast). However, we found an increased horizontal endpoint variability when the image-monitor contrast was low (Figure 8C), *F*(1,23) = 20.83, *p* < 0.001. Specifically, this was true in the object absent condition for the second, *t*(23) = 3.86, *p* < 0.001, and third saccade, *t*(23) = 2.24, *p* = 0.035. There was no significant interaction including the factor contrast.

### Discussion experiment 2

We measured initial saccadic endpoints and subsequent gaze positions for images containing one or no salient object. Critically, the contrast between image background and monitor background was either low or high. Whereas the initial orienting toward images was only affected by the presence or absence of objects and not by the contrast manipulation (Figure 6), a low image-monitor contrast was associated with a reduced gaze bias toward the image center for images containing no object (Figure 8). This was due to an increased (horizontal) endpoint variability (Figure 9).

These results suggest that the initial orienting toward images is primarily affected by the presence of salient objects in the images. Our setup favored early saccades by using a gap paradigm. In consequence, we observed a relatively high number of eye movements that were most likely made in anticipation of image onset (i.e., latency < 80 ms) and that were directed to the screen center. These early saccades could reflect a screen bias (Bindemann, 2010). However, we consider it more likely that these saccades reflect the spatial distribution of images and objects in our experiment. Whereas the screen center never spatially coincided with the location of an object or with the image center, it constituted the midpoint between the two relevant locations and might across all trials be a strategically advantageous location when starting to explore the images. The endpoints of saccades, particularly for larger latencies, were clearly affected by the images. Whereas endpoints in object present trials were increasingly directed toward the object location, endpoints in trials without object were increasingly biased toward the center of the image. For primary saccades, these tendencies started to saturate for saccade latencies of above 200 ms but were more pronounced at a later point in time for secondary saccades.

We did not find any evidence that the initial orienting toward images was affected by the image-monitor contrast (Figure 6). However, we observed a gaze bias toward the image center when the contrast between image background and monitor background was high (Figure 8). This gaze bias emerged approximately 950 ms after image onset and was present throughout the remaining exploration. This makes it unlikely that the differences in gaze position due to the contrast manipulation (starting after 950 ms) can be explained by boundary detection or region filling, which have been shown to start earlier (Poort et al., 2012; Self et al., 2013). Even an attentional modulation of figure-ground segregation can be found roughly 150 ms after image onset (Poort et al., 2012). Instead, the image-monitor manipulation affected the consistency of saccade target selection.

## General discussion

When humans shift their gaze toward images, the eyes predominantly land at or near the image center (e.g., Tatler, 2007; Bindemann, 2010). Here, we showed that this initial orienting toward the image center is not purely strategic, but that saccades in a certain time window were involuntarily biased toward the image center (Figure 3). This involuntary bias was measured as the deviation from (i) the instructed location, (ii) the screen midline, (iii) the only physically salient location in the image, and as the deviation from (iv) the only meaningful object in the image. This involuntary bias toward the image center thus exists beyond low-level salience, image semantics, or behavioral goals and most likely reflects center-of-gravity computations of the image outline. Yet, when the image center and a salient object directly compete, then the involuntary bias toward the salient object prevails over any involuntary bias toward the image center (Figure 3 & Figure 6). The initial orienting toward images was only affected by the presence or absence of salient objects, but not by the image-monitor contrast. However, in the absence of salient objects, a lower image-monitor contrast reduced the gaze bias toward the image center during the remaining exploration.

The present results provide further evidence that visual selection depends on time. The earliest saccades, that were possibly made in anticipation, were directed toward the average spatial location of stimuli (Figure 6). Saccades that were initiated in a time window between approximately 80 ms until up to 250 ms after image onset were prone to involuntary biases, either by salient stimuli or the image center (Figure 3). Later responses were reliably directed toward the instructed location and thus toward the behavioral goal (Figure 3). These results are consistent with a variety of previous findings (Ludwig & Gilchrist, 2002; van Zoest et al., 2004; Donk & van Zoest, 2008; Schütz et al., 2012; Wolf & Lappe, 2020; van Heusden et al., 2021). Without a behavioral goal, long-latency saccades are, just like short-latency saccades, primarily directed toward salient stimuli. Behavioral goals or task demands, on the other hand, can overwrite salience (Einhäuser, Rutishauser, & Koch, 2008; Schütz et al., 2012; Wolf et al., 2019; Wolf & Lappe, 2020). Similar to task demands overwriting salience, our results showed that salience or salient objects dominate over central fixation tendencies (Figure 3C & Figure 6). With our present experiments and stimulus material we cannot, however, distinguish whether this is due to low-level salience in the image (e.g., luminance contrast) or due to image semantics, because objects in the image were both the only salient and the only meaningful item. On the one hand, recent work suggests that semantics guides attention in natural scenes beyond central fixation tendencies (Peacock et al., 2020). On the other hand, the temporarily constricted bias toward a salient object observed in Experiment 1 is highly consistent with the bias measured toward a relatively meaningless luminance bar in earlier experiments (Wolf & Lappe, 2020). Yet, the latter only addresses the initial orienting toward stimuli after their appearance. Visual selection during subsequent image exploration might be more strongly related to image semantics (Nyström & Holmqvist, 2008; Henderson & Hayes, 2017; Peacock et al., 2020).

When investigating how low-level image properties or image semantics affect the visual exploration of images, the central fixation bias is often sought to be reduced to a minimum. A variety of temporal and spatial experiment settings have been proven helpful in reducing central fixation tendencies. First, avoiding a central pretrial fixation marker is helpful. Although the absence of an image-centered pretrial fixation cross does not eliminate the center bias (Tatler, 2007), it will make sure that gaze is not at the image center in the first place and can thus help to reduce central fixation tendencies. Second, reducing anticipatory and short-latency responses toward the image (Figure 3; Rothkegel et al., 2017) reduces central fixation tendencies. Short-latency saccades can be discouraged by using an overlap paradigm (removing the fixation cross after image onset; Fischer et al., 1997) or by introducing an additional go signal after image onset. If the image location as well as its temporal onset are predictable, anticipatory saccades often bring the line of sight to the anticipated image location or in between two anticipated locations (Figure 6). If the displayed images are smaller than the monitor on which they are displayed, anticipatory saccades can be minimized by additionally varying the image region. Third, a low contrast between image and monitor background can help to further reduce central fixation tendencies—at least in the absence of one clearly salient object. Images often have less homogeneous backgrounds and contain a variety of objects. Using a homogeneous monitor background might thus result in a low image-monitor contrast for one part of the image but not for other parts. Other manipulations (e.g., spatially pooling the monitor background from the nearby image information) might be more advised for more complex images and might in addition to luminance and color also retain information about orientation (at least for lower spatial frequencies). Yet, for homogenous image backgrounds, adjusting the monitor background can help to reduce central fixation tendencies.

## Supplementary Material

Supplement 1
