# Supplementary material for: Salient objects dominate the central fixation bias when orienting toward images
Source: J Vis. 2021 Aug 25;21(8):23. doi: 10.1167/jov.21.8.23 (PMC8399466; doi:10.1167/jov.21.8.23)
Supplement: Supplement 1 [file jovi-21-8-23_s001.pdf]

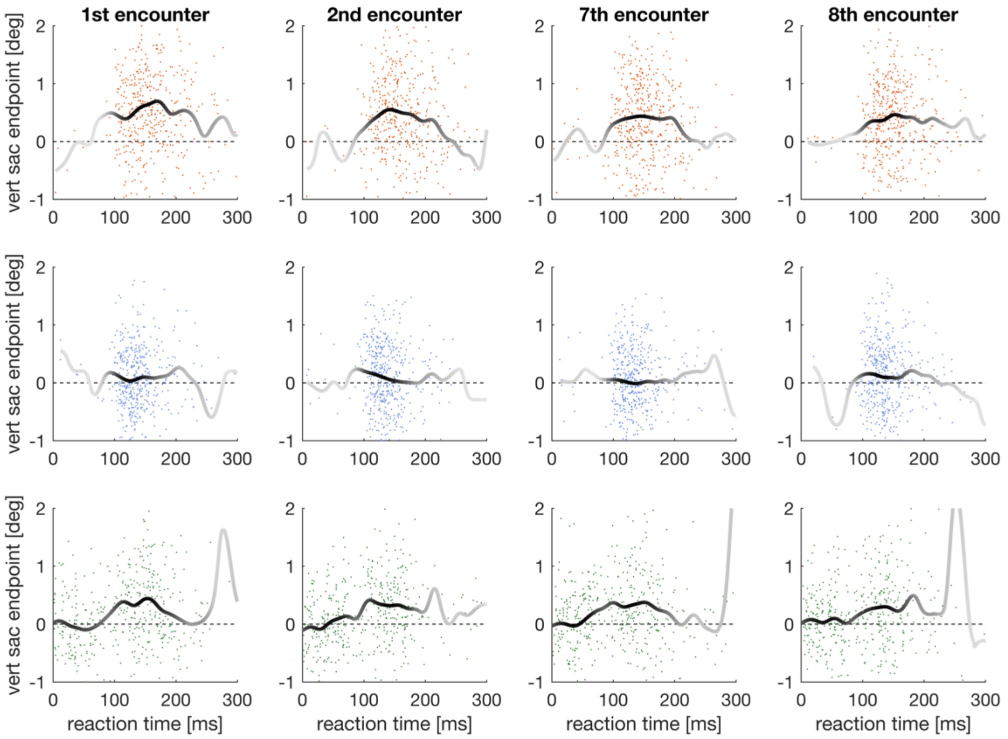

Suppl. Fig. S1. No evidence for a qualitative change with repeated image encounters in Experiment 1. Vertical saccade endpoints for the three respective conditions (upper row: *look at image center*, middle row: *look at object*, lower row: *look at cue*) for the first two (column 1 & 2) and the last two encounters with an image (column 3 & 4). Data points in each panel are endpoints from all participants on all images. The solid line represents a weighted average that was computed by means of a sliding Gaussian window with a standard deviation of 16 ms. The darker the line the more data points contribute to the estimate of that time point. Plotting conventions are identical to Figure 2D-F.

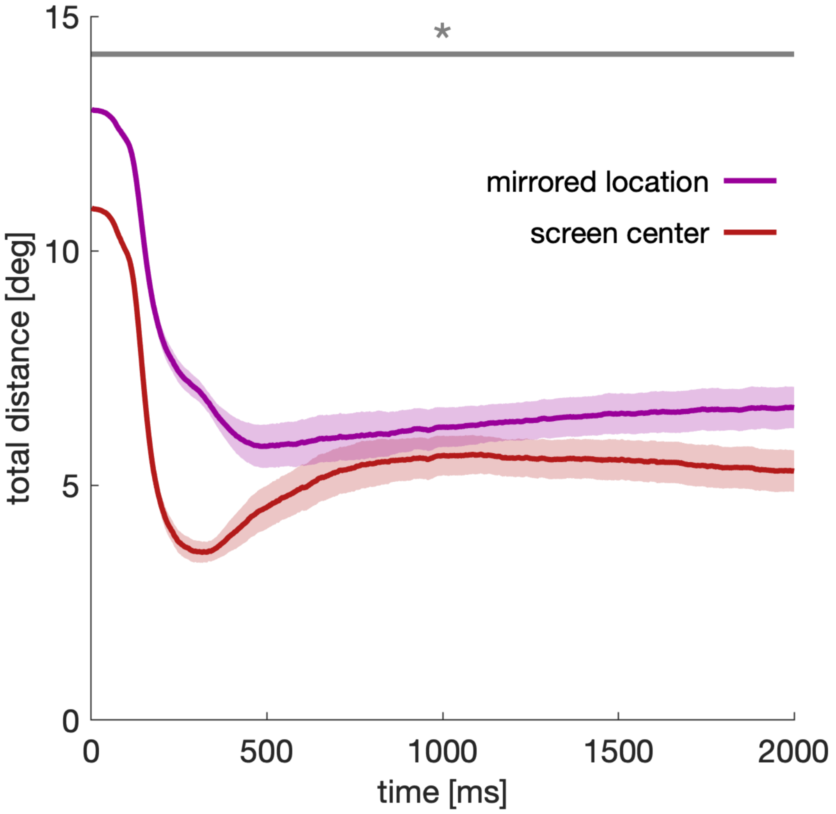

Suppl. Fig. S2. Distance to screen center over time in Experiment 2. For object absent trials, we compared the distance to the screen center (red curve) with the distance to the mirrored location (purple curve) with respect to image center. Thus, both locations were on the vertical image/screen midline and horizontally 3.52 deg away from the image center. We compared the time courses using a cluster-permutation analysis with 1000 permutations (see Methods of Experiment 2 for more details on this analysis). Gaze was closer to the screen center than to the mirrored location, $t = 25829$, $t_{crit} = 582.6$, $p < 0.01$, 1-2000 ms. Thus, gaze positions in the object absent condition were not symmetrically distributed around the image center but (always) biased towards the launch site. This finding can either be due to a bias towards the screen or due to a bias to remain close to the initial fixation position.
